# Molecular classification of endometrial carcinoma: clinical utility of an NGS panel with targeted detection of 116 cancer-related genes

**DOI:** 10.3389/pore.2026.1612465

**Published:** 2026-07-28

**Authors:** Lingfeng Chen, Zhijie You, Xunbin Yu, Yijuan Wu, Xin Chen, Jie Lin

**Affiliations:** Department of Pathology, Shengli Clinical Medical College of Fujian Medical University, Fujian Provincial Hospital, Fuzhou University Affiliated Provincial Hospital, Fuzhou, Fujian, China

**Keywords:** endometrial carcinoma, immunohistochemistry, molecular classification, NGS, Sanger sequencing

## Abstract

**Background:**

This study aimed to evaluate the efficacy of a single-test, targeted DNA next-generation sequencing (NGS) panel in classifying endometrial carcinoma (EC) into molecular subtypes and to compare its performance with that of the established Sanger sequencing + immunohistochemistry (Sanger + IHC) molecular classification.

**Methods:**

Targeted DNA NGS was performed on 131 samples using the clinically validated AmoyDx® Comprehensive Panel, and a commercially available targeted AmoyDx EC Panel covering POLE, TP53, and MSI was used for 63 samples.

**Results:**

The concordance between the NGS and Sanger + IHC classifications was 93.8% (182/194 cases), with a kappa value of 0.908. The exclusion of seven discordant *POLE* mutations improved concordance to 97.4% (kappa = 0.962). NGS identified 30 *POLE* mutations compared to 23 detected by Sanger sequencing, which missed low-frequency variants. Microsatellite instability (MSI) analysis and mismatch repair (MMR) immunohistochemistry (IHC) results were highly concordant (97.9%). However, NGS-based *TP53* mutation detection showed moderate agreement with the p53 IHC results (kappa = 0.688). Mutations associated with targeted therapy trials, including PTEN (76.3%), PIK3CA (50.4%), and ARID1A (35.9%), were found in 131 EC samples.

**Conclusion:**

These findings indicate that NGS-based molecular classification aligns well with Sanger + IHC molecular classification and offers higher sensitivity than Sanger sequencing, thereby improving the identification of mutations associated with targeted therapy trials. This enhances the prognosis and treatment planning for patients with advanced EC.

## Introduction

Endometrial carcinoma (EC) is a significant health concern, being the second most common malignant tumor of the female reproductive system in China and the leading gynecological cancer in developed countries [1]. Traditionally, the prognosis and treatment strategies for EC are guided by histopathological factors. However, recent advancements in molecular biology have underscored the importance of molecular classification in refining prognostic assessments and therapeutic decisions.

In 2013, The Cancer Genome Atlas (TCGA) project revolutionized our understanding of EC by introducing a molecular classification system based on comprehensive multi-omics analyses, including whole genome sequencing, whole exome sequencing, methylation profiling, transcriptomic analysis, and proteomics [2]. The TransPORTEC classification and Proactive Molecular Risk Classifier for EC (ProMisE) have also been developed. The ProMisE categories consist of four distinct molecular subtypes: POLE ultramutated (POLEmut), mismatch repair deficient (MMRd), p53 abnormal (p53abn), and non-specific molecular profile (NSMP) [3, 4]. These classifications have provided deeper insights into the heterogeneity of EC, revealing significant variations in the prognosis and response to treatment among EC subtypes.

Building on the TCGA framework, the 5th edition of the World Health Organization (WHO) classification of female genital tumors in 2020 incorporated molecular classification criteria, enabling direct clinical application through DNA sequencing and immunohistochemistry (IHC) techniques [5]. This integration into clinical guidelines is further supported by the 2023 staging recommendations from the International Federation of Gynecology and Obstetrics (FIGO), which advocates for comprehensive molecular classification testing for all EC patients [6]. Despite these advancements, the implementation of molecular classification in clinical practice remains inconsistent, primarily because of the complexity and cost associated with multi-platform testing methods, such as combining Sanger sequencing or next-generation sequencing (NGS) with IHC.

Approximately 3% of EC cases exhibit overlapping molecular characteristics, complicating the accurate determination of their molecular subtypes without comprehensive testing of all relevant molecular markers, including *POLE* mutations, MMR status, and p53 abnormalities [7]. The NSMP subtype, often referred to as the “junk category,” encompasses EC cases that do not exhibit *POLE* mutations, MMR deficiencies, or p53 abnormalities. This subtype accounts for a substantial proportion of EC cases, ranging from 30.2% to 47.4% in Western populations and even higher, between 47.7% and 67.6%, in Chinese populations [8]. The high prevalence and molecular heterogeneity within the NSMP subtype pose significant challenges, as it includes aggressive variants such as dedifferentiated or undifferentiated EC with *SMARCA4* loss, which have poor prognoses despite the absence of *TP53* mutations [9].

Recent studies have demonstrated excellent concordance between molecular classification methods such as ProMisE and NGS panels, highlighting the potential of NGS for the clinical molecular classification of EC [10, 11]. NGS offers superior sensitivity compared to traditional Sanger sequencing, particularly for detecting low-frequency *POLE* mutations, which are crucial for accurate subtype classification and subsequent treatment planning [12]. Moreover, NGS panels can simultaneously identify a wide array of mutations associated with targeted therapy trials, including alterations in genes such as *PIK3CA*, *ARID1A*, *BRCA1/2*, *PTEN*, *KRAS*, *AKT1*, *FBXW7*, and *HER2*, thereby providing valuable information for the prognosis and personalized therapy of patients with advanced EC.

This study aimed to evaluate the efficacy of a single-test targeted DNA NGS panel in classifying EC into molecular subtypes and compare its performance with that of the established Sanger sequencing + immunohistochemistry (Sanger + IHC) molecular classification. By implementing a clinically validated NGS panel, we sought to streamline the molecular classification process, enhance the sensitivity of mutation detection, and identify additional mutations that may inform the prognosis and therapeutic strategies for patients with advanced EC.

## Materials and methods

### Patient cohort, sample collection, and pathological information

This retrospective study included 196 patients with EC who underwent surgical resection at our hospital between January 2022 and December 2024. The patients did not receive neoadjuvant chemotherapy or radiotherapy prior to surgery. Formalin-fixed paraffin-embedded (FFPE) tumor samples were retrieved from the Pathology Department Biobank. Two patients were excluded because of sequencing failure or insufficient tumor tissue for DNA extraction, resulting in a final sample size of 194 patients with EC.

Two pathologists performed pathological classification, tumor grading, assessment of myometrial invasion, and lymphovascular space invasion according to the WHO guidelines [5]. Clinical staging was based on the International Federation of Gynecology and Obstetrics staging system (2023) [6]. This study was approved by the Ethics Committee of our hospital, and written informed consent was obtained from all participants.

### Immunohistochemistry

Each FFPE tissue sample was sectioned into 3.5 µm slices for IHC staining using the LUMATAS automatic pathological staining system. The primary antibodies used were from Fuzhou Maixin Biotech (Anti-MLH1, clone MX063; Anti-PMS2, clone MX073; Anti-MSH2, clone MX061; Anti-MSH6, clone MX056; Anti-p53, clone MX008). All histological and IHC slides were independently reviewed by two pathologists.

Normal protein expression of *MLH1*, *PMS2*, *MSH2,* and *MSH6* was defined as normal nuclear expression. Loss of expression was defined as the complete loss of nuclear expression, whereas internal controls (stromal or lymphocytic cells) exhibited strong nuclear staining. Based on the MMR protein expression status, tumors were categorized into two groups: deficient mismatch repair (dMMR) if any of the MMR proteins (*MLH1, PMS2*, *MSH2*, and *MSH6*) were lost, and proficient mismatch repair (pMMR) if all MMR proteins were positively expressed.

p53 expression patterns were classified as overexpression (OE), complete absence (CA), cytoplasmic expression (CY), or wild-type (WT), with OE, CA, and CY constituting abnormal p53 expression patterns. Tumors with p53 abnormalities (p53abn), if ≥75% of tumor cell nuclei exhibited strong or diffuse staining (OE), were completely negative (CA), or showed cytoplasmic staining (CY) [13, 14].

Abnormal subclonal expression was considered to occur in p53 and MMR proteins, if abnormal staining patterns were detected in areas adjacent to the tumor.

### DNA extraction

Paraffin-embedded tumor samples selected by pathological examination were subjected to DNA extraction using the MagPure FFPE DNA LQ Kit (Magen), according to the manufacturer’s protocol. Genomic DNA concentration was quantified using a NanoDrop ND-1000 spectrophotometer (Thermo Fisher Scientific).

### Sanger sequencing

*POLE* gene mutations were detected using Sanger sequencing, as previously described [15]. Exons 9–14 of the *POLE* gene were amplified using primers provided by Sangon Biotech (Shanghai) Co., Ltd. Only 11 previously identified pathogenic variants (P286R, V411L, S297F, S459F, A456P, F367S, L424I, M295R, P436R, M444K, and D368Y) were targeted [16]. PCR products were confirmed by gel electrophoresis, purified using the MinElute PCR Purification Kit (QIAGEN) according to the manufacturer’s instructions, and subsequently subjected to sequencing using the BigDye Terminator v3.1 Cycle Sequencing Kit (Applied Biosystems). Sequencing reactions were purified and analyzed using a SeqStudio Genetic Analyzer (Applied Biosystems). All *POLE* mutations were annotated according to the LRG_789 (NM_006231.3) and Human Genome Variation Society (HGVS) guidelines.

### Next-generation sequencing

Targeted DNA NGS was performed on 131 samples using the clinically validated AmoyDx® Comprehensive Panel (Amoy Diagnostics, Xiamen, China), which comprises 116 cancer-related genes, including 109 genes for single nucleotide variants (SNVs), insertions/deletions (indels), 31 genes for copy number variations (CNVs), and 12 genes for gene fusions. The panel covers mutations in *POLE, TP53, PTEN, PIK3CA, ARID1A, KRAS, HER2, BRCA1, BRCA2*, and *SMARCA4* and includes microsatellite instability (MSI) analysis. Genes and their detection regions are listed in Supplementary Table S1. MSI status was determined based on the percentage of unstable loci out of 55 microsatellite loci: samples with <15% unstable loci were classified as microsatellite stable (MSS), and those with ≥15% were classified as MSI-high (MSI-H). A commercially available targeted AmoyDx EC panel covering *POLE, TP53*, and MSI was used for 63 samples in this study (Amoy Diagnostics, Xiamen, China).

Sequencing was performed on an Illumina NextSeq 500 platform (Illumina) with the following quality criteria: Q30 base percentage ≥75%, coverage ≥95%, CoverageRatioUNIQ180 (proportion of hotspot regions with effective sequencing depth ≥180×) ≥95%, cnv_cv (coefficient of variation for sequencing depth within gene regions) <0.4, and cnv_uni (degree of deviation from the panel of normals) <1.5 (see Supplementary Tables S2, S3). The mutation allele frequency (MAF) thresholds for mutation identification by NGS were set as follows: SNVs and insertions/deletions (indels) ≥5% and CNVs ≥4 copies. Sequencing data were analyzed using bioinformatics software, and the therapeutic relevance of the identified genetic variants was interpreted based on publicly available databases, including the NCCN Guidelines and OncoKB [17]. The evidence levels for variant-drug associations were categorized into four tiers (A-D) according to the AMP/ASCO/CAP guidelines [18]: Tier A (FDA-approved or supported by professional clinical guidelines), Tier B (validated by large-scale clinical studies with expert consensus), Tier C (supported by Tier A evidence in other cancer types, used as inclusion criteria in clinical trials, or supported by multiple small-scale studies), and Tier D (preclinical studies or case reports).

### Bisulfite treatment and methylation-specific PCR

Bisulfite conversion of purified DNA was performed using the EpiTect Fast DNA Bisulfite Kit (Qiagen), following the manufacturer’s protocol. PCR products were analyzed using 3% agarose gel electrophoresis to verify successful conversion and amplification (Supplementary Figure S1).

### WHO molecular classification and NGS molecular classification

Molecular classification was performed according to the WHO guidelines [5]. Patients with one of the 11 confirmed pathogenic *POLE* variants detected by Sanger sequencing were categorized as POLEmut. The remaining EC cases were classified based on MMR status, with tumors showing loss of one or more MMR proteins designated as MMRd, and those with intact MMR protein expression classified as pMMR. Subsequently, EC cases were further stratified based on p53 status, where abnormal p53 expression patterns (OE, CA, and CY) were categorized as p53abn, and normal p53 expression (WT) was categorized as NSMP.

For NGS-based classification, patients with one of the 11 confirmed pathogenic *POLE* variants identified by the NGS panel were grouped as NGS POLEmut. The remaining EC cases were classified based on MSI status, with MSI-H cases categorized as NGS MSI-H/MMRd, and MSS cases further stratified based on *TP53* mutation status. Tumors harboring *TP53* mutations were designated NGS TP53mut/p53abn, whereas those without *TP53* mutations were classified as NGS NSMP/TP53wt.

### Statistical analysis

The consistency between Sanger + IHC and NGS molecular classifications was evaluated using overall accuracy and the kappa coefficient. Associations between molecular subgroups and clinicopathological characteristics were assessed using appropriate statistical tests, including the χ^2^ test for categorical variables and analysis of variance (ANOVA) for continuous variables. All statistical analyses were two-tailed, with a significance set at *P* < 0.05. Data analysis was performed using the IBM SPSS Statistics software (version 19.0; IBM Corp., Armonk, NY, USA).

## Results

### Descriptive statistics of EC patients

A total of 194 endometrial carcinoma specimens were successfully to molecular classification using NGS. The clinical and pathological characteristics of the patients are shown in Table 1. Based on the NGS molecular classification, 15.5% (30/194) of the cases were categorized as NGS POLEmut, 23.2% (45/194) as NGS MSI-H/MMRd, 14.4% (28/194) as NGS TP53mut/p53abn, and 46.9% (91/194) as NGS NSMP/TP53wt. Notably, the NGS classification identified 10.3% (20/194) of cases with multiple molecular features. Among these, ten NGS POLEmut tumors harbored *TP53* mutations, with one case exhibiting three distinct molecular features (POLEmut, MSI-H, and TP53mut), and ten NGS MSI-H/MMRd tumors concurrently displayed *TP53* mutations.

**TABLE 1:** Descriptive statistics of patients by NGS molecular classification subgroups based on post-operative specimens.

Patient characteristics	Total	NGS POLEmut	NGS MSI-H/MMRd	NGS TP53mut/p53abn	NGS NSMP/TP53wt	*p*-value
Age at surgery	​	​	​	​	​	0.006
Mean(SD)	58.4 (±9.7)	55.2 (±8.0)	60.8 (±8.6)	62.4 (±7.9)	57.1 (±10.6)	​
Median	58	56	58	62	57	​
Grade	​	​	​	​	​	<0.001
G1	60 (31.9%)	8 (27.6%)	10 (22.7%)	0 (0.0%)	42 (46.7%)	​
G2	82 (43.6%)	11 (37.9%)	22 (50.0%)	5 (20.0%)	44 (48.9%)	​
G3	46 (24.5%)	10 (34.5%)	12 (27.3%)	20 (80.0%)	4 (4.4%)	​
Histological subtype	​	​	​	​	​	<0.001
Endometrioid (EM)	170 (89.0%)	25 (83.3%)	40 (90.9%)	15 (57.7%)	90 (98.9%)	​
Clear cell	7 (3.7%)	2 (6.7%)	1 (2.3%)	4 (15.4%)	0 (0.0%)	​
Serous	6 (3.1%)	0 (0.0%)	0 (0.0%)	6 (23.1%)	0 (0.0%)	​
Mixed EM and serous	2 (1.0%)	2 (6.7%)	0 (0.0%)	0 (0.0%)	0 (0.0%)	​
Dedifferentiated	3 (1.6%)	1 (3.3%)	1 (2.3%)	0 (0.0%)	1 (1.1%)	​
Mixed EM and carcinosarcoma	1 (0.5%)	0 (0.0%)	0 (0.0%)	1 (3.8%)	0 (0.0%)	​
Mixed serous and carcinosarcoma	2 (1.0%)	0 (0.0%)	2 (4.5%)	0 (0.0%)	0 (0.0%)	​
Stage	​	​	​	​	​	0.135
I	124 (66.0%)	22 (73.3%)	31 (70.5%)	9 (37.5%)	62 (68.9%)	​
II	24 (12.8%)	2 (6.7%)	4 (9.1%)	5 (20.8%)	13 (14.4%)	​
III	35 (18.6%)	6 (20.0%)	8 (18.2%)	8 (33.3%)	13 (14.4%)	​
IV	5 (2.7%)	0 (0.0%)	1 (2.3%)	2 (8.3%)	2 (2.2%)	​
Myometrial invasion	​	​	​	​	​	0.043
None	18 (9.6%)	7 (23.3%)	4 (9.1%)	3 (12.5%)	4 (4.5%)	​
<50%	105 (56.1%)	17 (56.7%)	23 (52.3%)	10 (41.7%)	55 (61.8%)	​
≥50%	64 (34.2%)	6 (20.0%)	17 (38.6%)	11 (45.8%)	30 (33.7%)	​
LVSI	​	​	​	​	​	0.013
Yes	125 (67.2%)	22 (73.3%)	23 (52.3%)	12 (52.2%)	68 (76.4%)	​
No	61 (32.8%)	8 (26.7%)	21 (47.7%)	11 (47.8%)	21 (23.6%)	​
Lymph node involvement	​	​	​	​	​	0.592
Yes	163 (88.1%)	27 (90.0%)	37 (84.1%)	20 (83.3%)	79 (90.8%)	​
No	22 (11.9%)	3 (10.0%)	7 (15.9%)	4 (16.7%)	8 (9.2%)	​

NOTE: the total number of cases for each parameter may be less than the overall total because of missing data.

Abbreviations: LVSI, lymphovascular space invasion; POLEmut, POLE, ultramutated; MMRd, mismatch repair deficient; p53abn, p53 abnormal; NSMP, non-specific molecular profile. MSI-H, high microsatellite instability.

In comparison, the Sanger + IHC molecular classification assigned 11.9% (23/194) of cases to the POLEmut category, 23.7% (46/194) to MMRd, 13.9% (27/194) to p53abn, and 50.5% (98/194) to NSMP. The Sanger + IHC classification identified ten cases with overlapping molecular characteristics: five POLEmut tumors showed abnormal p53 expression or deficient mismatch repair (dMMR), and five MMRd tumors concurrently exhibited abnormal p53 expression.

### Comparison between NGS and sanger + IHC molecular classifications

**FIGURE 1:**
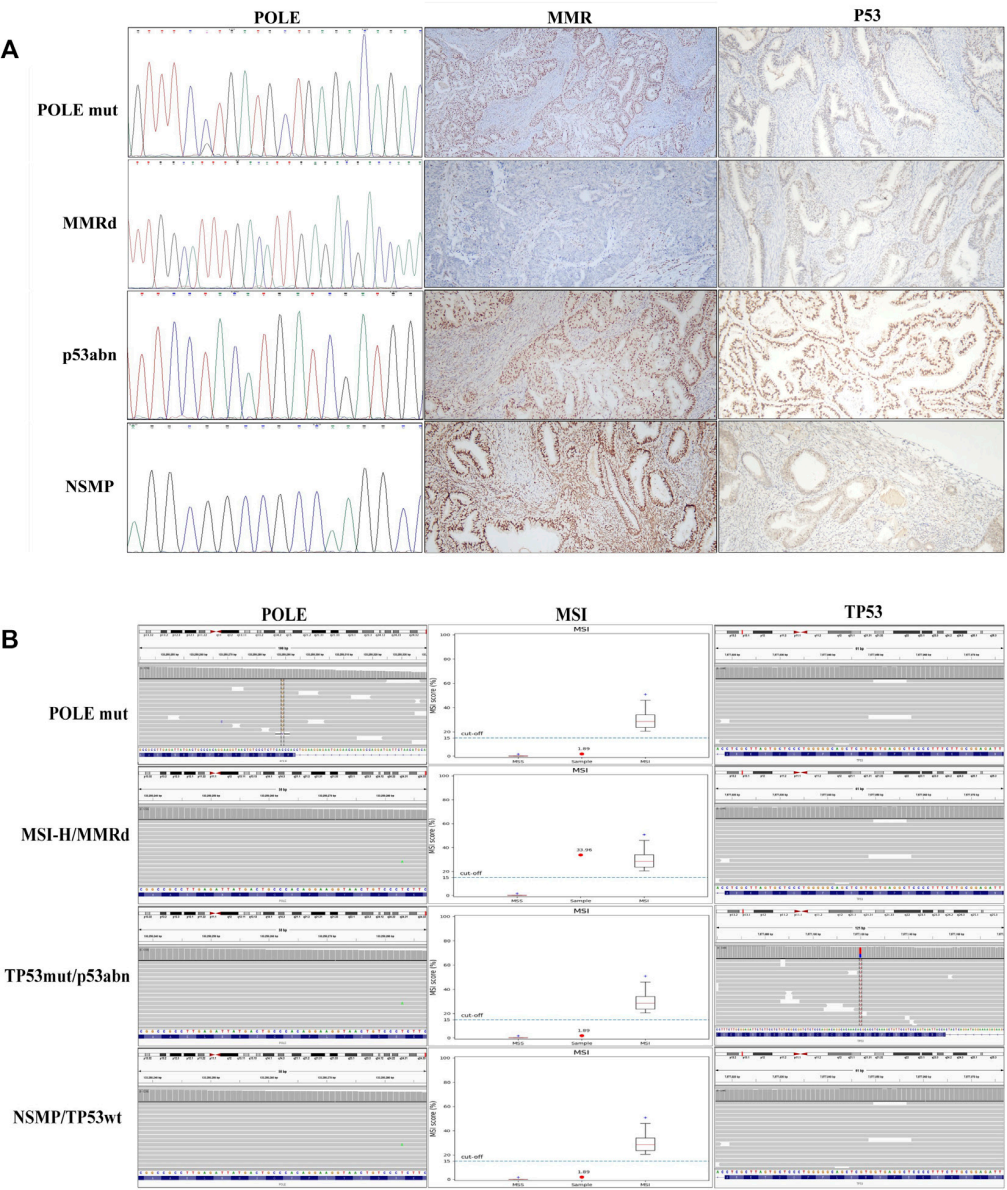
Representative images depicting Sanger + IHC and NGS molecular classification. **(A)** Sanger + IHC molecular classification (*POLE* mutation detected by Sanger sequencing and MMR and p53 mutations detected by IHC). **(B)** NGS molecular classification (detection of *POLE* mutations, MSI status, and *TP53* mutations using NGS). Abbreviations: POLEmut - POLE-Mutated; MMRd - Mismatch Repair-Deficient; p53abn - p53 abnormal; NSMP - No Specific Molecular Profile; MSI-H - high microsatellite instability; TP53mut - Tp53-Mutated; TP53wt - Tp53 wild-type.

**FIGURE 2:**
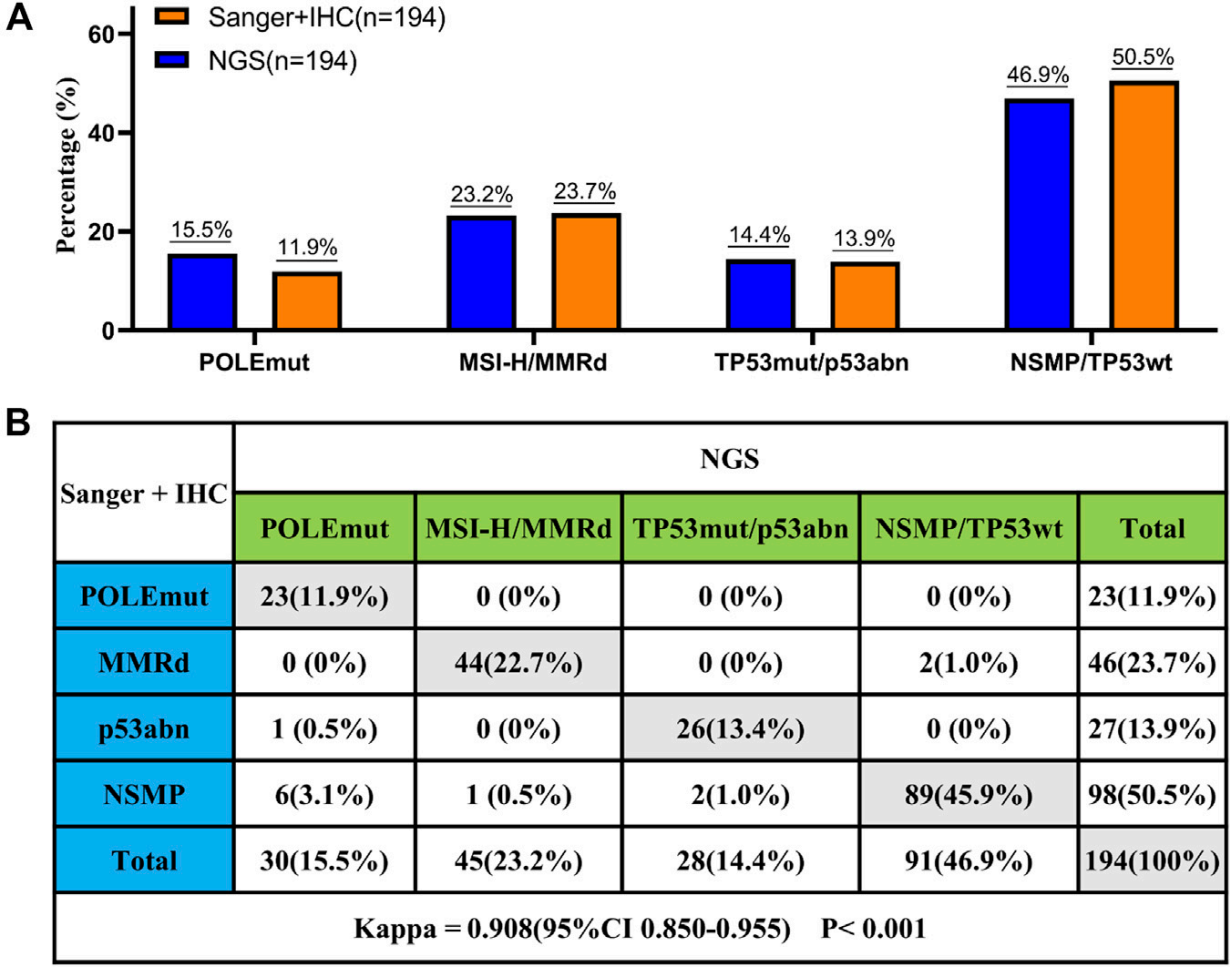
Molecular subtype distribution of prospectively accrued endometrial based on NGS and Sanger + IHC molecular classification. **(A)** This study included all 194 prospectively accrued endometrial carcinomas, and bar plots depict the molecular subtype distribution according to NGS and Sanger + IHC molecular classifications. **(B)** Concordance between NGS and Sanger + IHC molecular classifications.

**FIGURE 3:**
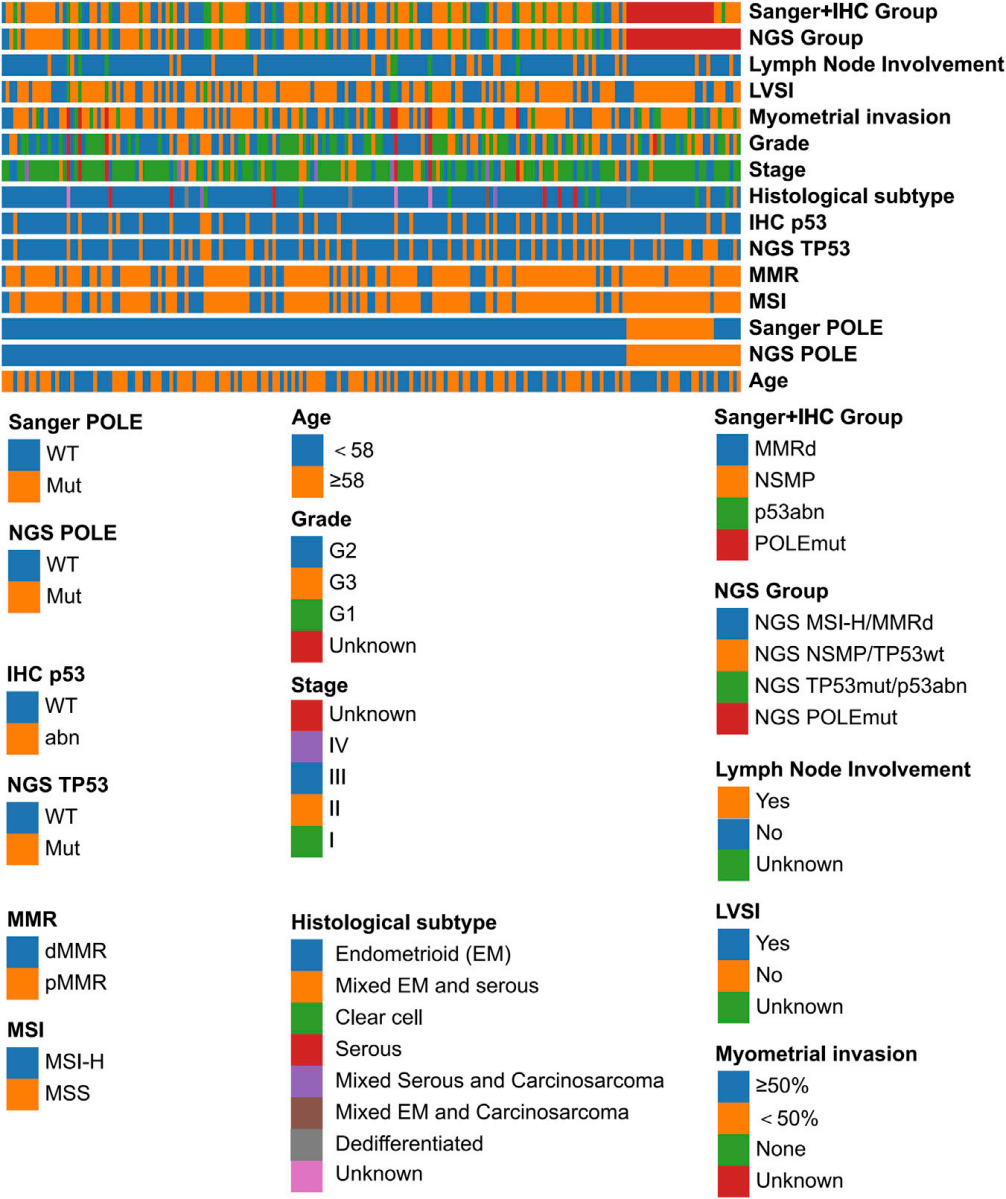
Oncoprint of genomic alterations in the four molecular subgroups of the cohort (n = 194). Endometrial cancers classified according to NGS or Sanger + IHC molecular classification are shown as the NGS and Sanger + IHC groups, respectively. Both NGS-based and Sanger-based *POLE* mutations are shown in the figure. Both NGS-based MSI status and IHC-based MMR are shown in the figure, as are the results of the NGS-based *TP53* mutations and IHC-based p53 assessment. Abbreviations: POLEmut - POLE-Mutated; MMRd - Mismatch Repair-Deficient; p53abn - p53 abnormal; NSMP - No Specific Molecular Profile; MSI-H - high microsatellite instability; TP53mut - Tp53-Mutated; TP53wt - Tp53 wild-type.

Figure 1 presents the molecular marker results for both Sanger + IHC and NGS classifications. A comparative analysis revealed that 93.8% (182/194) of the cases showed concordance between the NGS and Sanger + IHC molecular classifications, achieving a kappa coefficient of 0.908 and an overall accuracy of 0.938, (Figures 2, 3). However, there were 12 discordant cases between the two classification methods (Table 2). Specifically, cases 1 and 2 were classified as NSMP by Sanger + IHC but as NGS TP53mut/p53abn, with *TP53* variants S260Qfs*3 and G245S detected at a MAF of 2.4% and 15%, respectively. Both cases had tumor cell contents exceeding 50%, suggesting tumor heterogeneity, with a low proportion of *TP53*-mutated cells. Cases 3 and 4 were classified as MMRd by Sanger + IHC but as NGS NSMP/TP53wt, with no variants detected in the *MSH2, MSH6, MLH1, PMS2,* or *EPCAM* genes. Additionally, seven cases with *POLE* mutations were missed by Sanger sequencing because of their lower sensitivity than NGS, particularly for variants with a MAF <10%. After excluding the seven POLEmut cases that were missed by Sanger sequencing, the concordance between NGS and Sanger + IHC molecular classifications increased to 97.4% (189/194), with a kappa value of 0.962.

**TABLE 2:** Cases with discordance between original Sanger + IHC and NGS panel classifications.

Case	Sanger + IHC	NGS panel	Possible explanations for discordance
1	NSMP	NGS TP53mut/p53abn	It is speculated that the tumor is heterogeneous, and the proportion of tumor cells with a *TP53* gene mutation is very low
2	NSMP	NGS TP53mut/p53abn	
3	MMRd	NGS NSMP/TP53wt	Due to MLH1 promoter methylation
4	MMRd	NGS NSMP/TP53wt	
5	NSMP	MSI-H/MMRd	False-positive staining may occur in cases of amino acid substitutions that lead to loss of function with preserved immunoreactive protein expression
6	NSMP	NGS POLEmut	Difference in detection sensitivity between NGS and sanger sequencing technology
7	NSMP	NGS POLEmut	
8	NSMP	NGS POLEmut	
9	NSMP	NGS POLEmut	
10	NSMP	NGS POLEmut	
11	NSMP	NGS POLEmut	
12	NSMP	NGS POLEmut	

### Consistency between NGS and sanger sequencing for *POLE* detection

**FIGURE 4:**
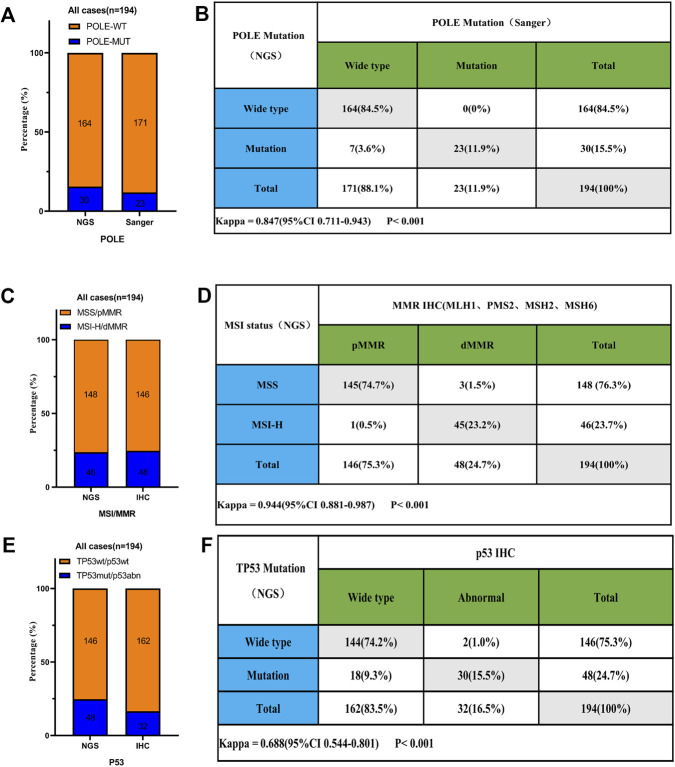
Comparison between NGS, Sanger sequencing, and immunohistochemistry. Bar plots show the concordance between **(A)** NGS and Sanger sequencing technology for *POLE* mutation analysis; **(B)** quantitative analysis of *POLE* mutations using NGS and Sanger sequencing technology; **(C)** NGS-based MSI status detection and MMR Immunohistochemistry; **(D)** quantitative analysis of NGS-based MSI status and MMR Immunohistochemistry and **(E)** NGS-based *TP53* mutation analysis and p53 immunohistochemistry. Table of concordance between **(F)** quantitative analysis of NGS-based *TP53* mutations and p53 immunohistochemistry.

**FIGURE 5:**
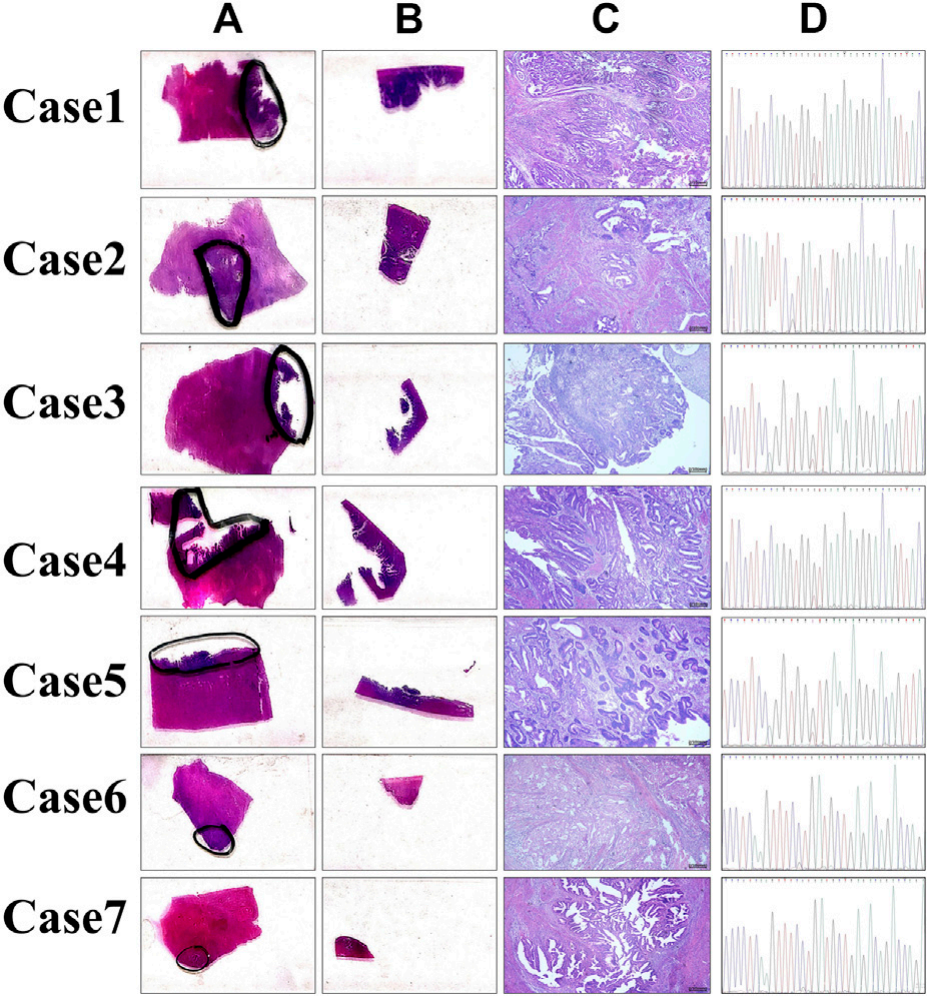
FFPE enrichment and resequencing of endometrial cancers were performed in 7 cases that were not detected by Sanger sequencing for POLE. **(A)** Seven original endometrial cancer samples with low tumor content. **(B)** HE staining was used to mark the tumor area and enrich the tumor content. **(C)** Enriched endometrial cancer tumor cell morphology. **(D)** *POLE* mutation detected by Sanger sequencing.

As shown in Figure 4, NGS identified 30 *POLE* gene mutations, including P286R in 15 cases, V411L in 11 cases, A456P in two cases, and single instances of S297F and P436S. In contrast, Sanger sequencing detected only 23 *POLE* mutations, excluding seven cases with allelic frequencies <10% (Supplementary Table S4). These seven cases, which had low tumor content (20%), underwent tumor region delineation and enrichment based on hematoxylin and eosin (HE) staining prior to DNA extraction and subsequent Sanger sequencing. Remarkably, all seven cases were confirmed to harbor *POLE* mutations (Figure 5).

### Concordance between NGS-based MSI status and IHC-based MMR status

IHC for MLH1, MSH2, MSH6, and PMS2 proteins, along with PCR-based MSI analysis, are the standard methods for assessing MSI phenotypes in EC molecular classification. The NGS-based MSI status, determined by analyzing the read distribution across 55 microsatellite loci, was compared with the IHC-based MMR status. The results demonstrated high concordance, with 97.9% (190/194) of cases being consistent between the two methods. Four discordant cases were observed: three IHC-diagnosed dMMR cases were classified as MSS by NGS, and one IHC-diagnosed pMMR case was classified as MSI-H by NGS. These three cases exhibited concurrent loss of MLH1 and PMS2 expression and *MLH1* promoter hypermethylation (Supplementary Figure S1). Additionally, two cases displayed partial loss of MMR protein expression: one with a 50% loss of MLH1/PMS2, and the other with a 20% loss of MLH1/PMS2, indicating a subclonal staining pattern (Supplementary Figure S2), and both were positive for *MLH1* promoter methylation.

Among the 194 patients, 45 had MSI-H. Owing to tumor-only testing, the germline mutation status remained uncertain. Although the NGS panel also detected *MLH1, PMS2, MSH2,* and *MSH6* gene variants, no pathogenic MMR-related gene mutations were identified in the dMMR tumors. No mutations in *EPCAM* were detected in the 194 patients. Germline testing for Lynch syndrome is typically recommended. However, no mutations were detected in this cohort.

### Concordance between NGS-based *TP53* mutation detection and IHC-Based p53 status

Both NGS and IHC were used to determine p53 status in all EC cases in this study. Abnormal p53 status was defined by IHC as ≥75% of tumor cell nuclei exhibiting strong or diffuse staining, complete absence of nuclear staining, or cytoplasmic staining. Three cases had p53 expression levels near the threshold; 60%, 65%, and 70% of tumor cells showed moderate positive expression, and were thus classified as p53 wild-type (WT) according to the interpretation criteria.

NGS detected at least one potentially pathogenic *TP53* mutation in 48 of the 194 cases, including variants such as V73Wfs*50, C141Y, R181C, R213*, C238Y, S241F, G245S, R248W, R248Q, T253A, L257R, ES260Qfs*3, R267W, R273C, R273H, P278S, R282W, and E285Gfs*20. Additionally, two novel *TP53* variants (R202_V203del and V157_M160del) were identified using NGS, both of which were associated with mutant p53 expression levels.

Figure 4 highlights that 18 cases with *TP53* mutations detected by NGS were classified as p53 wild-type by IHC, resulting in a moderate concordance with a kappa coefficient of 0.688 and an overall concordance rate of 89.7% (174/194 cases). When POLEmut and MSI-H/MMRd cases were excluded, in which *TP53* mutations were often considered passenger mutations that did not influence the classification, the consistency between IHC-based p53 staining and NGS-based *TP53* mutation detection improved significantly. Among the remaining 119 cases, only three showed discordances, leading to an overall concordance rate of 97.5% (116/119 cases), as presented in Table 3.

**TABLE 3:** Comparison of molecular subtype diagnoses by NGS and IHC for p53.

NGS panel	p53 IHC		
	NSMP	p53abn	Total
NSMP/TP53wt	91 (76.5%)	0 (0%)	91 (76.5%)
TP53mut/p53abn	3 (2.5%)	25 (21.0%)	28 (23.5%)
Total	94 (79.0%)	25 (21.0%)	119 (100%)
Kappa = 0.927 (95% CI 0.830–1.000) P < 0.001			

Subclonal p53 expression (defined as mutant-pattern staining confined to discrete tumor foci occupying <10% of the total tumor area) was observed in 10/194 (5.2%) cases (Supplementary Figure S3). TP53 mutations were detected in 5/10 cases, all of which met the NGS detection threshold (MAF≥5%). Among these 10 tumors, five were molecularly classified as either mismatch repair deficient (MMRd, n = 1) or polymerase epsilon-mutated (POLEmut, n = 4). Notably, all five MMRd/POLEmut tumors predominantly exhibited wild-type p53 expression patterns with only focal mutant-pattern staining. TP53 mutations were identified in 4/5 cases, suggesting potential discordance between molecular and immunohistochemical (IHC) results due to either low tumor cellularity in sampled regions or subclonal mutations below the IHC detection threshold.

### Mutations associated with targeted therapy trials detected by NGS

**FIGURE 6:**
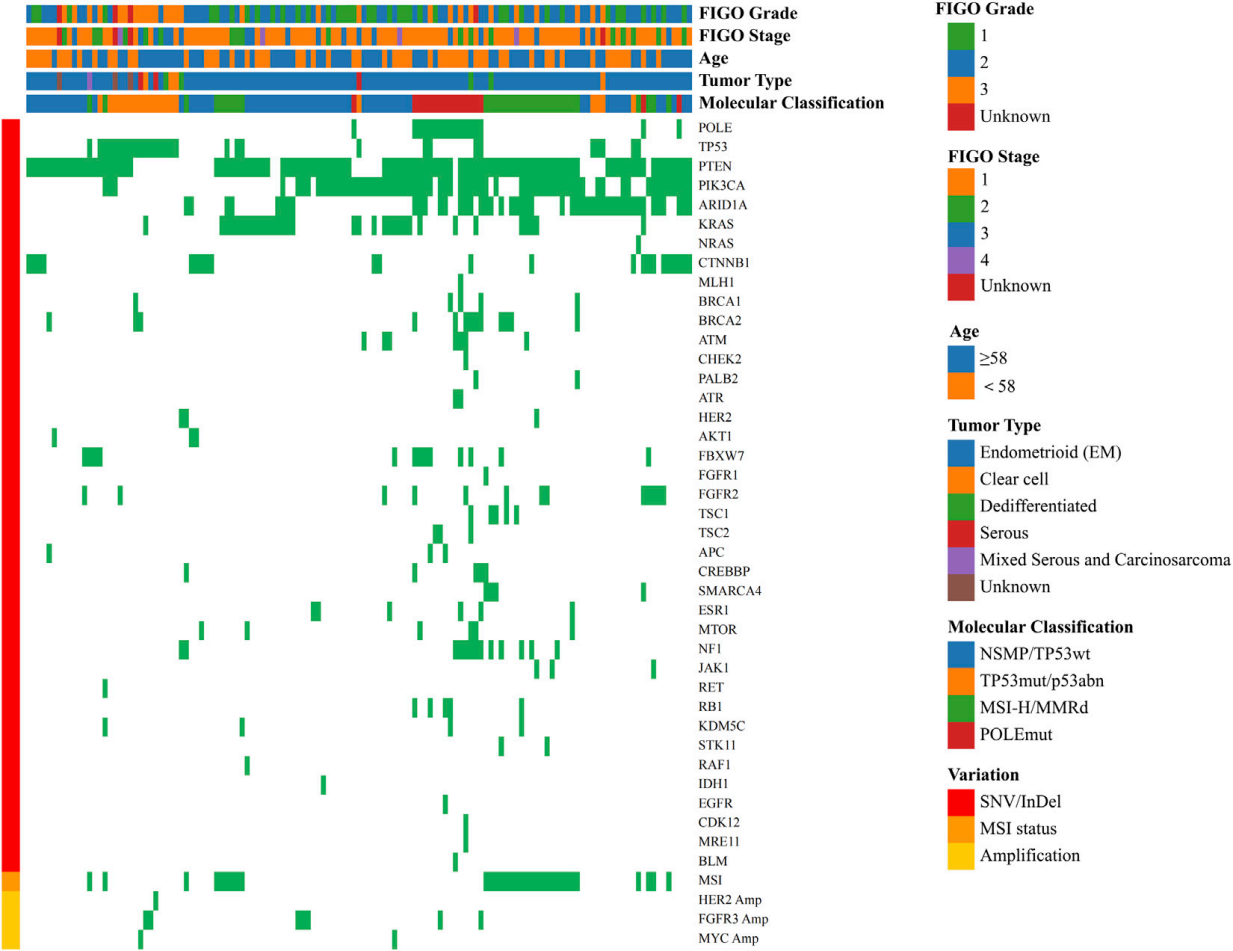
Repertoires of genetic alterations across histological subtypes of prospectively acquired endometrial cancer. Mutations and gene amplification were identified by sequencing, targeting 116 cancer-related genes in 131 prospectively accrued endometrial cancer cases. The cases are shown in columns, and the genes in rows. Tumor type, molecular classification, and variations are color-coded according to the legend.

Forty-two mutations associated with targeted therapy trials were identified in 131 samples (Figure 6). The most frequently mutated genes were *PTEN* (76.3%), *PIK3CA* (50.4%), and *ARID1A* (35.9%). Beyond the primary molecular markers *POLE*, *TP53*, and MSI, additional mutations with frequencies exceeding 10% included *KRAS* (25.2%) and *CTNNB1* (17.6%).

The distribution of specific gene mutations varied significantly across the FIGO stages (Table 4). For example, *PTEN* mutations decreased from 86.8% in FIGO G1 to 59.3% in FIGO G3 (P < 0.05), whereas *PIK3CA* mutations decreased from 60.5% in G1 to 29.6% in G3 (P < 0.05). Conversely, *TP53* mutations increased markedly from 2.6% in G1 to 59.3% in G3 (P < 0.001) and *HER2* mutations increased from 0% in G1 to 11.4% in G3 (P < 0.05).

**TABLE 4:** Statistical analysis of gene alterations associated with targeted therapy trials in 131 prospectively accrued endometrial cancer cases.

Patient characteristics		PTEN	*p* value	PIK3CA	*p* value	ARID1A	*p* value	KRAS	*p* value	CTNNB1	P value	TP53	*p* value	HER2	*p* value
​	Total	100 (76.3%)	​	66 (50.4%)	​	47 (35.9%)	​	33 (25.2%)	​	23 (17.6%)	​	30 (22.9%)	​	4 (3.1%)	​
Grade	​	​	0.036	​	0.043	​	0.132	​	0.110	​	0.085	​	<0.001	​	0.027
G1	38	33 (86.8%)	​	23 (60.5%)	​	13 (34.2%)	​	14 (36.8%)	​	8 (21.1%)	​	1 (2.6%)	​	0	​
G2	61	47 (77.0%)	​	32 (52.5%)	​	27 (44.3%)	​	14 (23.0%)	​	14 (23.0%)	​	9 (14.8%)	​	1 (1.6%)	​
G3	27	16 (59.3%)	​	8 (29.6%)	​	6 (22.2%)	​	4 (14.8%)	​	1 (3.7%)	​	16 (59.3%)	​	3 (11.4%)	​
Age	​	​	0.018	​	0.667	​	0.507	​	0.573	​	0.313	​	0.073	​	0.431
<58	58	50 (86.2%)	​	28 (48.3%)	​	19 (32.8%)	​	16 (27.6%)	​	8 (13.8%)	​	9 (15.5%)	​	1 (1.7%)	​
≥58	73	50 (68.5%)	​	38 (52.1%)	​	28 (38.4%)	​	17 (23.3%)	​	15 (20.5%)	​	21 (28.8%)	​	3 (4.1%)	​
Stage	​	​	0.031	​	0.464	​	0.110	​	0.288	​	0.195	​	0.031	​	0.003
I-II	104	83 (79.8%)	​	54 (51.9%)	​	41 (39.4%)	​	25 (24.0%)	​	21 (20.2%)	​	19 (18.3%)	​	1 (1.0%)	​
III-IV	23	14 (60.9%)	​	10 (43.5%)	​	5 (21.7%)	​	8 (34.8%)	​	2 (8.7%)	​	8 (34.8%)	​	3 (13.0%)	​

Age-related differences were also notable; patients aged ≥58 years exhibited significantly higher frequencies of *TP53* mutations (28.8% vs. 15.5%, *P* > 0.05), *CTNNB1* mutations (20.5% vs13.8%, *P* > 0.05), and *HER2* mutations (4.1% vs. 1.7%, *P* > 0.05) than those aged <58 years.

In advanced EC (FIGO Stage III-IV), mutations in *PTEN* (60.9% vs. 79.8% in early stages, P < 0.05), *PIK3CA* (43.5% vs. 51.9%, *P* > 0.05), *ARID1A* (21.7% vs. 39.4%, *P* > 0.05), *CTNNB1* (8.7% vs. 20.2%, *P* > 0.05), *FBXW7* (0% vs. 12.5%, *P* > 0.05), and *FGFR2* (4.3% vs. 11.5%, *P* > 0.05) were significantly less frequent than in early-stage disease. Conversely, *HER2* mutations were significantly more frequent in patients with advanced EC (13.0% vs. 1.0%, *P* < 0.01).

Histological subtype-specific findings revealed that all four clear-cell EC cases harbored *TP53* mutations, as did both serous EC cases, with one also exhibiting *HER2* amplification, and the other presenting a *BRCA1* mutation alongside MYC amplification. Among the four dedifferentiated EC cases, one had POLEmut, one had dMMR, one had p53abn, and one had NSMP with concurrent *HER2* and *NF1* mutations. Additionally, one case of mixed carcinoma (serous and sarcoma) was classified as a dMMR.

## Discussion

This study confirmed that a single-test, DNA-based NGS panel for molecular classification of EC achieves high concordance with the multi-platform Sanger + IHC molecular classification, resolving Sangers low sensitivity for low-frequency POLE mutations. By addressing the discrepancies caused by the lower sensitivity of Sanger sequencing in detecting *POLE* mutations, the consistency between NGS and Sanger + IHC classifications improved significantly, with discordance observed in only 2.6% of cases (5 out of 194). These findings confirm and refine prior evidence on NGS-based EC molecular classification and align with previous research by Huvila et al. [10], who reported a high agreement rate between the ProMisE molecular classification and the FoundationOne NGS panel, and by Li et al. [11], who found high concordance between an 11-gene NGS panel and the ProMisE classification in 70 cases.

A key practical advancement is resolving limitations in POLE mutation detection: Sanger sequencing fails to identify low-abundance POLE mutations in low-tumor-content samples, risking misclassification of POLEmut as p53abn/MMRd and overtreatment. Currently, there is no validated immunohistochemical surrogate for pathogenic POLE mutations suitable for routine clinical use. This represents one of the main limitations of the WHO molecular classification and poses a challenge in clinical practice. The accuracy of both NGS and Sanger sequencing was significantly affected by the tumor cell content. In this study, NGS detection of genes, such as *POLE,* covered CDS and approximately 20 base pairs of the exon-intron junction, with a mutation frequency >1%, capable of detecting key low-frequency pathogenic variants. In contrast, Sanger sequencing can only detect variants with a frequency >20% under routine conditions [19]. Owing to the detection threshold, there is a risk of missing cases that are limited to the uterus, clinically early, with small tumors and low tumor cell proportions. When paraffin tumor samples have less than 20% tumor cell content, it can affect the detection of low-abundance *POLE* gene mutations, such as the seven cases in this study where NGS detected *POLE* gene mutations (V411L or P286R) with mutation frequencies of 7.2%, 6.9%, 5.1%, 4.4%, 4.2%, 4.2%, and 3.2%, respectively, but Sanger sequencing failed to detect these *POLE* variations. After tumor tissue separation and enrichment to ensure that the tumor content was above 20%, Sanger sequencing detected *POLE* mutations in all seven cases. This indicates that when paraffin tumor samples are not quality-controlled for tumor cell content by HE staining, Sanger sequencing may miss some cases of *POLE* mutations, incorrectly classifying POLEmut as the p53abn or MMRd type, leading to erroneous prognostic predictions and overtreatment. Therefore, when the tumor cell content in a sample is low, it is crucial to match the HE-stained cancer foci, perform tumor tissue separation and enrichment, and ensure the highest quality and accuracy of the selected samples, particularly for tumors with low-frequency *POLE* mutations. Therefore, we recommend NGS over Sanger sequencing for detecting *POLE* mutations.

Furthermore, multiple studies have underscored the reliability of NGS in assessing MSI status compared with IHC-based MMR protein detection [20–22]. Similarly, the concordance between NGS-based *TP53* mutation detection and IHC-based p53 status has been explored, revealing a moderate agreement [13, 23]. These studies highlight the potential of NGS for the clinical molecular classification of EC, offering a streamlined and highly sensitive approach.

In this study, the concordance between NGS-based MSI status and IHC-based MMR status was high (97.9%), with four discordant cases. The three IHC-diagnosed dMMR cases were classified as microsatellite stable (MSS) by NGS, which was attributed to low tumor purity, insufficient MSI loci in the panel, bioinformatic threshold, subclonal MMR deficiency and *MLH1* promoter hypermethylation, a phenomenon previously reported [24], indicating that *MLH1* methylation can lead to the loss of protein expression without detectable MMR gene mutations. Additionally, two patients with partial loss of MMR protein expression also exhibited *MLH1* promoter hypermethylation, reinforcing the association between epigenetic modifications and MMR deficiency. One case, initially classified as pMMR by IHC, was reclassified as MSI-H based on the NGS analysis. This discordance may be explained by retained immunoreactivity despite loss of protein function, which can occur when amino acid substitutions lead to the functional loss of MMR proteins while maintaining their immunoreactivity. Such cases highlight the limitations of IHC alone in accurately assessing MMR status and underscore the importance of integrating molecular testing for precise classification.

The evaluation of p53 status revealed moderate concordance (kappa = 0.688) between NGS-based *TP53* mutation detection and IHC-based p53 expression. The use of a less commonly used antibody clone (MX008) for p53 IHC staining may limit comparability of immunohistochemical results with those from other similar cohorts. Notably, most discrepancies occurred within the POLEmut and MMRd subtypes, where *TP53* mutations are often passenger mutations that do not influence the molecular classification. After excluding these subtypes, the agreement between the NGS and IHC methods improved dramatically to 97.5%, underscoring the importance of sequential molecular classification steps in improving diagnostic precision. The remaining discordant cases warrant further investigation because they pose clinical challenges for treatment decision-making. The emergence of novel *TP53* mutations such as V157_M160del and R202_V203del highlights the need for ongoing research to elucidate their functional and clinical implications.

Notably, this study reinforces the translational value of NGS beyond molecular classification: the detection of mutations associated with targeted therapy trials (including *PTEN, PIK3CA, ARID1A, KRAS, CTNNB1,* and *HER2*) and prognostic markers (including *KRAS* and *CTNNB1*) aligns with emerging precision oncology data. These findings are crucial for guiding targeted therapies and improving prognostic assessment in patients with advanced EC. For instance, mutations in PI3K/AKT pathway genes (e.g., *PIK3CA* and *PTEN*) are actionable targets for inhibitors such as alpelisib and capivasertib [25–27]. Similarly, *HER2* amplifications identified in p53 abnormal (p53abn) EC subtypes can inform the use of *HER2*-targeted therapies such as trastuzumab, which has been incorporated into clinical guidelines for *HER2*-positive, advanced, or recurrent serous EC [28, 29]. Additionally, the detection of *BRCA1/2* mutations opens avenues for PARP inhibitor therapy, which has shown efficacy in *BRCA*-mutated cancers [30, 31].

The NSMP subtype, characterized by its molecular heterogeneity, includes tumors with aggressive features such as *KRAS* and *CTNNB1* mutations. Identifying these mutations within the NSMP group provides valuable prognostic information and underscores the need for further risk stratification. For example, *KRAS* mutations in combination with wild-type *ARID1A* have been associated with poorer disease-free survival [32], whereas *CTNNB1* mutations are linked to reduced progression-free survival in younger patients with early stage EC [33]. These insights emphasize the role of comprehensive molecular profiling in refining the prognosis and tailoring individualized treatment strategies.

Despite the clear advantages of NGS-based molecular classification, there are inherent limitations to this approach. The retrospective nature, limited cohort size, and single-center design of this study may affect the generalizability of our findings to a broader population. Additionally, the absence of long-term clinical follow-up and prognostic data necessitates further prospective studies to validate the clinical utility of NGS-based molecular classifications for EC; we cannot confirm NGS-based molecular classifications therapeutic predictive utility.

While this study aligns with and builds on existing literature, we provide actionable, practice-ready refinements: the single-test DNA-based NGS panel is a sensitive, streamlined alternative to Sanger + IHC for EC molecular classification and resolves POLE detection failures. Additionally, the NGS panel identified numerous mutations associated with targeted therapy trials, enhanced prognosis, and enabled personalized treatment strategies for patients with advanced EC. These findings support the integration of NGS-based molecular classification into clinical practice to improve the diagnostic precision and treatment outcomes.

## Data Availability

The datasets presented in this study can be found in online repositories. The names of the repository/repositories and accession number(s) can be found in the article/Supplementary Material.

## Supplementary material

SUPPLEMENTARY FIGURE S1 Methylation-specific PCR. Representative examples of MSP analysis of MLH1 methylation. (U) Lanes represent amplification of unmethylated alleles, and (M) lanes contain only methylated alleles (Lane M represents a 100-bp DNA marker).

SUPPLEMENTARY FIGURE S2 Subclonal MMR immunostaining. Immunostaining for MLH1 and PMS2 in cases 50 and 122, respectively, revealed a subclonal staining pattern.

SUPPLEMENTARY FIGURE S3 Subclonal p53 immunostaining. **(A,B)** Cases showing a combination of normal (wild-type) and p53abn overexpression. **(C,D)** Cases showing a combination of normal (wild-type) and complete absence.
