# Supplementary material for: Molecular classification of endometrial carcinoma: clinical utility of an NGS panel with targeted detection of 116 cancer-related genes
Source: Pathol Oncol Res. 2026 Jul 28;32:1612465. doi: 10.3389/pore.2026.1612465 (PMC13457160; doi:10.3389/pore.2026.1612465)
Supplement: Supplementary file 1 [file Supplementaryfile1.docx]

**Table S1. NGS Molecular Classification Kit: Genes List and Detection Regions**

| **Gene** | **Nucleotide ID** | **Variation Type** | **Detection Regions** |
| --- | --- | --- | --- |
| *AKT1* | NM_001382430 | SNVs; indels | EX4,5 |
| *AKT2* | NM_001626 | CNV; SNVs; indels | EX2,4,6,8,10,12,14 |
| *AKT3* | NM_005465 | CNV; SNVs; indels | EX1,3,5,7,9,11,13 |
| *ALK* | NM_004304 | Fusion; SNVs; indels | EX18-28; IVS18,19 |
|  |  |  | EML4 (NM_019063) EX6,13,20; IVS6,13,20 |
| *APC* | NM_000038 | SNVs; indels | EX16 |
| *AR* | NM_000044 | SNVs; indels | EX4,5,8 |
| *ARAF* | NM_001654 | SNVs; indels | EX7,14 |
| *ARID1A* | NM_006015 | SNVs; indels | EX3,11,12,13,20 |
| *ATM* | NM_000051 | SNVs; indels | EX17,47,59,63 |
| *ATR* | NM_001184 | SNVs; indels | EX10 |
| *AURKA* | NM_198437 | CNV; SNVs; indels | EX2-9 |
| *BAP1* | NM_004656 | SNVs; indels | All Exons |
| *BCL2L11* | NM_138621 | SNVs; indels | EX2,3,4,IVS2 |
| *BRAF* | NM_004333 | Fusion; SNVs; indels | EX8,9,11,15; IVS8 |
| *BRCA1* | NM_007294 | SNVs; indels | All Exons |
| *BRCA2* | NM_000059 | SNVs; indels | All Exons |
| *CCND1* | NM_053056 | CNV; SNVs; indels | EX1-5 |
| *CCNE1* | NM_001238 | CNV; SNVs; indels | EX2,4,6,8,10,12 |
| *CD274* | NM_014143 | CNV; SNVs; indels | EX2-7 |
| *CDK12* | NM_016507 | SNVs; indels | EX1,2,14 |
| *CDK4* | NM_000075 | CNV; SNVs; indels | EX2-8 |
| *CDK6* | NM_001145306 | CNV; SNVs; indels | EX2-8 |
| *CDKN2A* | NM_000077 | SNVs; indels | EX1-3 |
| *CDKN2B* | NM_004936 | SNVs; indels | EX1-2 |
| *CREBBP* | NM_004380 | SNVs; indels | EX16,25-27,30,31 |
| *CTNNB1* | NM_001904 | SNVs; indels | EX3 |
| *DDR2* | NM_006182 | SNVs; indels | EX5,8,13-18 |
| *EGFR* | NM_005228 | SNVs; indels; CNV | EX1-21 |
| *EIF1AX* | NM_001412 | SNVs; indels | EX1-7 |
| *EPAS1* | NM_001430 | CNV; SNVs; indels | EX1,3,5,7,9,11,13,15 |
| *EPCAM* | NM_002354 | SNVs; indels | All Exons |
| *ERBB2* | NM_004448 | CNV; SNVs; indels | EX1-27 |
| *ERBB3* | NM_001982 | SNVs; indels | EX18-24 |
| *ERBB4* | NM_005235 | SNVs; indels | EX18-24 |
| *ESR1* | NM_000125 | SNVs; indels | EX5,7,8 |
| *ETS2* | NM_005239 | SNVs; indels | EX8 |
| *FANCA* | NM_000135 | SNVs; indels | EX33 |
| *FBXW7* | NM_001349798 | SNVs; indels | EX4-14 |
| *FGF19* | NM_005117 | CNV; SNVs; indels | All Exons,IVS2 |
| *FGF3* | NM_005247 | CNV; SNVs; indels | EX1-3,IVS2 |
| *FGFR1* | NM_023110 | Fusion; CNV; SNVs; indels | EX2,4,6,8,10,12,14,16-18; IVS17;3’-UTR |
| *FGFR2* | NM_000141 | Fusion; CNV; SNVs; indels | EX2,4,6,8,10,12,14,16-18; IVS17;3’-UTR |
| *FGFR3* | NM_000142 | Fusion; CNV; SNVs; indels | All Exons; IVS17; 3'-UTR |
| *FGFR4* | NM_213647 | SNVs; indels | All Exons |
| *FLCN* | NM_144997 | SNVs; indels | EX4,9,11 |
| *FLT3* | NM_004119 | SNVs; indels | EX6 |
| *GNAS* | NM_000516 | SNVs; indels | EX1,8,9 |
| *HIF1A* | NM_001530 | CNV; SNVs; indels | EX1,3,5,7,9,11,13,15 |
| *HRAS* | NM_005343 | SNVs; indels | EX2,3 |
| *IDH1* | NM_005896 | SNVs; indels | EX4 |
| *IDH2* | NM_002168 | SNVs; indels | EX4 |
| *IGFIR* | NM_000875 | CNV; SNVs; indels | EX1,3,5,7,9,11,13,15,17,19,21 |
| *JAK1* | NM_002227 | SNVs; indels | EX12-25 |
| *JAK2* | NM_004972 | SNVs; indels | EX12-25 |
| *JAK3* | NM_000215 | SNVs; indels | EX11-24 |
| *KDM5C* | NM_004187 | SNVs; indels | All Exons |
| *KDR* | NM_002253 | SNVs; indels | EX21 |
| *KIT* | NM_000222 | SNVs; indels | EX9,11,13,14,17 |
| *KRAS* | NM_004985 | SNVs; indels | EX2,3,4 |
| *MAP2K1* | NM_002755 | SNVs; indels | EX1,2,6 |
| *MAPK1* | NM_002745 | CNV; SNVs; indels | EX1-8 |
| *MET* | NM_000245 | CNV; SNVs; indels | All Exons; IVS13-14 |
| *MLH1* | NM_000249 | SNVs; indels | All Exons |
| *MRE11* | NM_005591 | SNVs; indels | EX14 |
| *MSH2* | NM_000251 | SNVs; indels | All Exons |
| *MSH6* | NM_000179 | SNVs; indels | All Exons |
| *MTOR* | NM_004958 | SNVs; indels | EX29-31,39,40,42-45,47,48,50,53,56,57 |
| *MYC* | NM_002467 | CNV; SNVs; indels | EX1-3 |
| *NF1* | NM_001042492 | SNVs; indels | All Exons |
| *NF2* | NM_000268 | SNVs; indels | All Exons |
| *NOTCH1* | NM_017617 | SNVs; indels | EX2,6,34 |
| *NRAS* | NM_002524 | SNVs; indels | EX2,3,4 |
| *NRG1* | NM_013964 | Fusion | CD74(NM_001025159) EX7,8,9;IVS6,7,8 |
|  |  |  | SDC4 (NM_002999) EX2,3; IVS2 |
| *NTRK1* | NM_002529 | Fusion; SNVs; indels | EX8,9,10,13; IVS8,9 |
| *NTRK2* | NM_006180 | Fusion; SNVs; indels | EX10,11,13,14; IVS10,13 |
| *NTRK3* | NM_001012338 | Fusion; CNV; SNVs; indels | EX3,5,7,9,11,13,15,17,20 |
|  |  |  | ETV6 (NM_001987) EX4,5,6; IVS4,5 |
| *PALB2* | NM_024675 | SNVs; indels | All Exons |
| *PAX8* | NM_003466 | Fusion; SNVs; indels | EX8,9,10,11; IVS8,10 |
| *PDCD1* | NM_005018 | SNVs; indels | EX1-5 |
| *PDGFRA* | NM_006206 | CNV; SNVs; indels | EX2,4,6,8,10,12-14,16,18,19,21,23 |
| *PGR* | NM_000926 | CNV; SNVs; indels | EX1-8 |
| *PIK3CA* | NM_006218 | CNV; SNVs; indels | All Exons |
| *PIK3R1* | NM_181523 | SNVs; indels | EX9-11,13-16 |
| *PMS2* | NM_000535 | SNVs; indels | All Exons |
| *P OLD1* | NM_002691 | SNVs; indels | EX8,9,23 |
| *POLE* | NM_006231 | SNVs; indels | EX1-14 |
| *PSMD4* | NM_002810 | CNV; SNVs; indels | EX1,3,5,7,9 |
| *PTCH1* | NM_000264 | SNVs; indels | EX1,3,14,15 |
| *PTEN* | NM_000314 | SNVs; indels | All Exons |
| *RAF1* | NM_002880 | SNVs; indels | EX7,10-17 |
| *RASA1* | NM_002890 | SNVs; indels | EX9,16,20,21 |
| *RASAL1* | NM_001301202 | SNVs; indels | EX11 |
| *RB1* | NM_000321 | SNVs; indels | All Exons |
| *RET* | NM_020975 | Fusion; SNVs; indels; CNV | EX6,7,8,10-18; IVS6,7,10,11 |
| *RICTOR* | NM_152756 | CNV; SNVs; indels | EX1,3,5,7,9,11,13,15,17,19,21,23,25,27,29,31,33,35,37 |
| *RIT1* | NM_006912 | SNVs; indels | All Exons |
| *ROS1* | NM_002944 | Fusion; SNVs; indels | EX31-42; IVS31,33,34,35 |
|  |  |  | CD74(NM_001025159) EX7-9; IVS6,7,8 |
|  |  |  | SLC34A2(NM_006424)EX4,14;IVS4 |
|  |  |  | SDC4(NM_002999)EX2,3;IVS2 |
| *RSF1* | NM_016578 | CNV; SNVs; indels | EX1,3,5,7,9,11,13,15 |
| *SF3B1* | NM_012433 | SNVs; indels | EX14,15 |
| *SMD4* | NM_005359 | SNVs; indels | All Exons |
| *SMARCA4* | NM_003072 | SNVs; indels | All Exons |
| *SMO* | NM_005631 | CNV; SNVs; indels | All Exons |
| *STK11* | NM_000455 | SNVs; indels | All Exons |
| *TERT* | NM_198253 | SNVs; indels | Promoter Region |
| *TOP2A* | NM_001067 | CNV; SNVs; indels | EX1,3,5,7,9,11,13,15,17,19,21,23,25,27,29,31,33,35 |
| *TP53* | NM_000546 | SNVs; indels, SNP | All Exons, rs1042522 |
| *TSC1* | NM_000368 | SNVs; indels | All Exons |
| *TSC2* | NM_000548 | SNVs; indels | All Exons |
| *TSHR* | NM_000369 | SNVs; indels | EX10 |
| *VHL* | NM_000551 | SNVs; indels | All Exons |
| MSI | / | / | / |

Abbreviations: SNVs, single nucleotide variants; indels, insertions/deletions; CNVs, copy number variations; EX, exon; IVS, intervening sequence.

**Table S2. Standard of NGS Quality control**

| **Sample type** | **Quality control content** | **Quality control standard** |
| --- | --- | --- |
| DNA quality assessment | Total DNA | ≥100 ng |
|  | Total Library | ≥500 ng |
| Tumor tissue | Clean Q30 | ≥75％ |
|  | Coverage | ≥95％ |
|  | Coverage Ratio UNIQ180 | ≥95％ |
|  | cnv_cv | ＜0.4 |
|  | cnv_uni | ＜1.5 |

**Table S3. Threshold of NGS Site filtering**

| **Sample type** | **Quality control content** | **Threshold** |
| --- | --- | --- |
| Tumor tissue | sensitivity | SNV/Indel: 5%, CNV: 4 copies |
|  | Indel: ≥6bp | Var_US≥5, Var_SS≥1 |
|  | SNV/Indel (<6bp) HotSpot | Freq_US≥2%, Var_US≥5, Freq_SS≥1%, Var_SS≥2 |
|  | SNV/Indel (<6bp) non-HotSpot | Freq_US≥2.5%, Var_US≥13, Freq_SS≥1%, Var_SS≥8, Var_DS≥3 |
|  | HotFusion HotSpot | Var_US≥10 |
|  | HotFusion non-HotSpot | Var_US≥24，Var_SS≥16 |
|  | CNV | ≥5 |
|  | MSI | MSI_numerical≥15% |

**Table S4. Cases with discordant results between Sanger Sequencing and NGS Panel**

| Case | POLEmut | MAF |
| --- | --- | --- |
| 1 | V411L | 7.16% |
| 2 | P286R | 6.92% |
| 3 | V411L | 4.40% |
| 4 | V411L | 4.24% |
| 5 | V411L | 3.24% |
| 6 | P286R | 5.14% |
| 7 | V411L | 4.23% |
